# Support for the feasibility of the ages and stages questionnaire as a developmental screening tool: a cross-sectional study of South African and Zambian children aged 2-60 months

**DOI:** 10.1186/s12887-017-0802-3

**Published:** 2017-02-16

**Authors:** Alastair van Heerden, Celia Hsiao, Beatrice Matafwali, Julia Louw, Linda Richter

**Affiliations:** 1 Human Sciences Research Council, 750 Ridge Road, Durban, 4000 South Africa; 2 MRC/Wits Developmental Pathways for Health Research Unit, University of the Witwatersrand, Johannesburg, South Africa; 3 School of Education, The University of Zambia, Lusaka, Zambia; 4 DST-NRF Centre of Excellence in Human Development, University of the Witwatersrand, Johannesburg, South Africa

**Keywords:** Ages and stages questionnaire, Early child development, Southern Africa, South Africa, Zambia

## Abstract

**Background:**

There is a growing global acknowledgement that improving child survival rates is no longer sufficient. Emphasis is shifting to the improvement of health and developmental trajectories in early childhood. Screening and measurement of these trajectories in low and middle income countries is difficult, however, as they currently rely on developmental tests standardised among populations of children growing up in resource rich environments.

**Methods:**

This paper presents a comparison of one such tool adapted for use with children living in Southern Africa to children from the United States, Norway, Korea and Spain. The Ages and Stages Questionnaire version 3 (ASQ-3) was adapted and administered to 853 children living in South Africa and Zambia.

**Results:**

Children in southern Africa were found to perform significantly better than children from other countries early in life, especially in the domains of communication, gross motor and fine motor skills. By the age of five, children in southern Africa were performing significantly worse than their peers in the domains of fine motor and problem-solving.

**Conclusion:**

The results indicate the applicability of the ASQ-3 in southern Africa and point to the importance of early interventions to protect the early good development of African children in order to promote positive life trajectories.

## Background

The past two decades have seen phenomenal gains in child survival. Child deaths have been halved [1], from an estimated global average under-5 mortality rate of 84.6 per 1000 live births in 1990, to an average of 44.0 per 1000 in 2013 [2]. Even though the picture is less bright for Sub-Saharan Africa, which accounts for all ten countries with the highest under-5 mortality rate in 2013, rates fell consistently between 1.7 and 3.5% per year from 1970 to 2013. Despite these gains, it is estimated that more than 200 million children in low- and middle-income countries do not reach their developmental potential due to the impact of poverty and undernutrition on social, cognitive and physical development [3].

Improved child survival comes with costs that must be addressed. The 2011 WHO world report on disability found an estimated 1 in 20 children with disabilities globally in 2013 [4] and a near doubling of the prevalence of disability in lower income countries (18.0%) compared to higher income countries (11.8%); children (0–14 years)(4) bear the brunt [5].

Without intervention, multiple sources of risk can constrain the life-course trajectory of millions of children in Sub-Saharan Africa. As a result, there is growing interest from both government and non-governmental organisations in supporting the early detection and remediation of developmental delays. Providing such a service to children in resource-limited communities is, however, a challenge due to the likelihood that few if any developmental tests have been carefully translated, adapted from their high-income country origins and evaluated, and that will most likely be administered by paraprofessional health workers [6–8].

One tool which has garnered significant attention for use in low- and middle-income countries is *The Ages and Stages Questionnaire*, a developmental screening assessment standardised for use among children in the U.S. between the ages of 2 months and 5 years [9]. Relying on parent-report, the age-graded screener assesses children’s progress in each of five domains: fine motor, gross motor, communication, problem solving, and personal-social. Reliability and validity of the instrument has been well established in the United States [10, 11]. It has subsequently been standardised and validated for cross-cultural use in many parts of the world including Brazil [12], Taiwan [13], Iran [14], Spain [15], Canadian First Nations [16] and India [17]. Although variation in results is reported across these studies, they all broadly concur that the ASQ can be successfully applied in community-based, low-resource settings. As such, the ASQ may be an effective tool for identifying at-risk children in low- and middle-income countries. Unfortunately, to date, no work exists to establish the feasibility of administration or psychometric properties of the ASQ used among children living in sub-Saharan Africa.

This study was conducted for the purpose of cultural adaptation, validation and standardization of the ASQ questionnaire for 2–60-month-old children living in southern Africa. The psychometric analysis of the ASQ was conducted by Hsiao et al. [18] (under review). This paper updates and extends the work of Vameghi et al. [14] by comparing ASQ norms for Southern African children to those obtained from children in the US, Norway, Korea and Spain.

## Methods

### Population

Despite South Africa being classified as a middle-income country, 56% of children were estimated in 2012 to live in households with monthly per person incomes below the poverty line of R635 (US$59) [19]. In addition, in 2012, South Africa had 6.1 million people living with HIV, 408 000 of whom are children [20]. Despite economic growth and social transformation stunted growth among young children remains a persistent problem in South Africa. Recent stunting prevalence data suggest that around 26.9% of boys and 25.9% of girls under 3 years of age are stunted [21]. Zambia with an estimated population of just over 15 million people is defined by the World Bank as a lower-middle income country with a GDP of $27.07bl (23). Despite this classification, poverty as defined on the percentage of people living on less than $1.25 per day, has been slowly rising from an estimate of 68.5% in 2006 to 74.3% in 2010 (23). As a consequence, about 5% of young children in Zambia are thought to be severely malnourished, 16% are estimated as underweight and 45% to be stunted. From within this context, 853 typically developing and healthy children and their caregivers in Zambia and South Africa were randomly selected to participate in the study. Children from each country were sampled from communities with characteristics matched closely to those described above. The total sample consisted of 853 children. Of these 431 (50.5%) were from Zambia with 50.1% of the sample made up of girls. In South Africa, 422 (49.5%) children were enrolled (50% girls). All children were between the ages of 2 months and 5 years with approximately 10 boys and 10 girls in each of the 21 age groups of the ASQ-3 across the two countries. Children’s caregiver was mostly the child’s biological mother (85.6%).

### Recruitment

In South Africa, caregivers and children were recruited randomly from well-baby clinics and daycare centres in the rural area of Vulindlela, KwaZulu-Natal. The same recruitment procedure was used in Zambia with caregiver-children dyads recruited randomly from well-baby clinics, daycare centres and home visits in peri-urban settlements close to Lusaka. Locally, well-baby clinics are so named as they provide promotive and preventive services such as growth monitoring, immunization and treatment of minor childhood illnesses. Criteria for inclusion in the study were that the child was born full-term and normal birth weight, had no known disabilities or impairments, and no specific concerns about their child’s development were expressed by the parent or primary caregiver. Where available, parent information was validated with the Road-to-Health clinic cards. The Research Ethics Committee of the Human Sciences Research Council (HSRC) in South Africa, and the University of Zambia (UNZA) Humanities and Social Sciences Research Ethics Committee approved the study protocol (South Africa: REC 4/18/09/13, Zambia: IRB 00006464).

### Procedure

Appropriate consent and approval to approach caregivers and their children was first obtained from relevant government departments, preschools, day-care centres and community stakeholders. Caregivers were then approached at the facilities where the study was first explained and then informed consent sought. Research assistants were recruited and trained in South Africa and Zambia. All were female with at least some tertiary training and experience of working with children. Training consisted of three phases. In the first phase, group work was used to review and address any confusion about item meaning. If necessary, the item was clarified and specific actions demonstrated to ensure that the item would be conveyed correctly to the caregiver (e.g., “Without giving your child help by pointing or using gestures, ask him to ‘put the book on the table’ and ‘put the shoe under the chair.’ Does your child carry out both of these directions correctly?”). Secondly, an interaction was simulated by inviting four caregivers and their children with a range of ages to participate in a pilot at each site so that research assistants could have the opportunity to practice the study procedure - from obtaining informed consent to administering the questionnaires, and answering any questions that caregivers may have. The last phase of training moved research assistants out of the classroom and into the field. During this piloting phase research assistants gained experience of using the tools and materials in the field. During each of these phases, the team held regular debriefing meetings to discuss any challenges faced and lessons learnt. For instance, research assistants were video recorded during the pilot phase and video clips were played back so that the group could identify issues and discuss potential solutions. In Zambia, mothers who were invited to participate in the study, many of whom had never been exposed to research studies in the past, turned to their traditional leaders in the community for guidance. Once the study began, research assistants were supported through continued supervision and regular group meetings. In these sessions, discussions were held with respect to data collection challenges in order to ensure the quality of the data by addressing issues as they arose.

### Analyses

Raw scores of the standardization sample were converted to Z-scores in order to construct a normal distribution. These normalised scores were used to calculate descriptive statistics of performance in each age interval as well as cut-off scores for the study sample. Cut-off scores were determined based on a score of two standard deviations below the mean within each domain across each age interval. As only summary statistics (mean, sample size and standard deviation) were available for each of the comparison countries and population variance not available or unknown, Welch’s *t*-test was conducted using the formula

$$ t=\frac{{\overline{X}}_1-{\overline{X}}_2}{{}^S\overline{X}_1-{\overline{X}}_2}\kern0.5em \mathrm{where}\ {}^S\overline{X}_1-{\overline{X}}_2=\sqrt{\frac{S_1^2}{n_1}+\frac{S_2^2}{n_2}.} $$

The test statistic approximates an ordinary Student's t distribution with the degrees of freedom calculated using the Welch–Satterthwaite equation.

## Results

### Sample and mean scores

As reported by Hsiao et al. [18], the children’s caregiver was found most frequently to be the child’s biological mother (85.6%). Other caregivers who were familiar with the children’s development included aunt or uncle (6%), grandparent (4.7%), biological father (1.3%), or other (2.4%). Table 1 presents further demographic characteristics of the study sample as described in Hsiao et al. [18] (under review). Cronbach’s alpha and item-total correlations were calculated to examine the internal consistency of the ASQ-3 across the 21 ages. Cronbach’s alpha ranged from .09 (personal-social domain at 16 months) to .79 (gross fine-motor domain at 60 months). For the total score, Cronbach’s alpha ranged from .60 (42 months) to .88 (24 months). Between 7.9 and 14.3% of the items in each domain were found to have an item-correlation lower than 0.30.

**Table 1:** Demographic characteristics of study sample N (%)

	South Africa	Zambia	Combined	*p*-value
Caregiver level of education				
Completed primary school	32 (7.6)	124 (28.8)	156 (18.3)	***
Completed secondary school	357 (84.6)	244 (56.6)	601 (70.5)	***
Completed tertiary education	33 (7.8)	63 (14.6)	96 (11.3)	***
Caregiver employment				
Yes	59 (14)	107 (24.8)	166 (19.5)	***
No	363 (86)	322 (74.7)	685 980.3)	***
Child attending preschool/daycare				
Yes	140 (33.2)	54 (12.5)	194 (22.7)	***
No	282 (66.8)	377 (87.5)	659 (77.3)	***
Household SES (Mean)^a^	10.58	10.39	10.48	ns

^a^Assessed based on sum of assets owned from a list of 22 items

Table 2 presents the summary table for all domains across all measured time points and their interpretation, which is covered extensively by Hsiao et al.[18] (under review). Table 3 extends the work of Vameghi et al.[14] by including the results for Southern African children into the original comparison of the United States, Norway, Korea and Spain. Although the sample size of 853 caregiver child dyads in this study is smaller than in the comparison countries, age-specific variance was found to be comparable. The results from Table 3 are summarised graphically in Table 4, highlighting trends that emerge from this multifaceted comparison. Results from these three tables are presented together by domain. In general, boys and girls did not differ in performance across any of the age intervals and developmental domains. The only exception was that girls scored higher than boys in both the gross motor domain at 8 months, and the problem solving domain at 9 months. Consequently, results are not disaggregated by gender. To maintain an adequate sample size results are also not disaggregated by country and all figures represent combined results for South Africa and Zambia.

**Table 2:** Number of participants, means, standard deviations and cut off scores for each domain, M(SD) as presented in Hsiao et al. [18] (under review)

Age	n	Communication	Cut off	Gross Motor	Cut off	Fine Motor	Cut off	Problem Solving	Cut off	Personal-Social	Cut off
2	42	52.98 (9.94)	33.09	55.49 (6.96)	41.56	56.10 (5.76)	44.58	45.71 (12.08)	21.56	41.19 (8.10)	34.99
4	38	57.25 (5.66)	45.94	58.59 (4.13)	50.33	52.63 (9.47)	33.68	52.00 (11.37)	29.26	52.89 (8.43)	36.03
6	41	54.39 (7.51)	39.36	58.59 (4.13)	50.33	53.17 (9.60)	33.98	50.00 (10.78)	28.44	42.07 (12.04)	17.99
8	40	56.13 (5.83)	44.47	55.00 (8.16)	38.67	57.50 (6.20)	45.10	52.00 (12.75)	26.50	53.25 (9.23)	34.78
9	42	51.31 (11.48)	28.35	41.67 (13.28)	15.10	53.21 (6.70)	39.81	49.05 (11.44)	26.17	40.83 (13.02)	14.80
10	40	55.25 (8.98)	37.28	50.50 (10.61)	29.28	55.13 (5.90)	43.32	52.75 (8.69)	35.36	48.38 (12.32)	23.74
12	40	55.50 (6.68)	42.14	51.38 (13.2)	24.97	44.63 (10.58)	23.46	49.50 (11.65)	26.21	43.88 (14.70)	14.48
14	40	48.13 (9.85)	28.42	49.50 (14.93)	19.65	41.5 (14.06)	13.38	48.75 (10.79)	27.18	44.25 (11.07)	22.11
16	40	47.63 (12.30)	23.03	53.00 (10.49)	32.02	50.50 (11.37)	27.76	51.50 (10.51)	30.47	52.75 (7.68)	37.40
18	42	44.76 (12.79)	19.17	52.86 (12.10)	28.65	49.02 (12.61)	23.80	46.38 (10.80)	24.77	53.33 (7.38)	38.57
20	41	46.10 (15.39)	15.32	54.15 (9.35)	35.45	44.15 (12.14)	19.87	40.73 (11.16)	18.42	53.66 (9.15)	35.35
22	41	41.46 (16.40)	8.66	54.27 (10.16)	33.95	40.73 (11.59)	17.54	48.45 (12.17)	24.08	49.39 (11.68)	26.02
24	39	50.38 (12.42)	25.53	54.00 (11.39)	31.22	44.13 (13.58)	16.96	45.75 (13.04)	19.68	49.36 (10.83)	27.69
27	41	51.71 (10.16)	31.38	53.78 (9.27)	35.24	43.41 (13.89)	15.63	45.50 (11.02)	23.45	42.80 (12.04)	18.72
30	40	49.15 (11.17)	26.80	55.87 (9.40)	37.08	45.12 (15.59)	13.94	41.71 (13.26)	15.39	46.87 (9.32)	28.23
33	39	53.75 (8.90)	35.95	57.75 (5.77)	46.21	44.10 (13.66)	16.78	41.71 (13.26)	15.39	46.87 (9.32)	28.23
36	41	55.12 (7.70)	39.71	56.59 (7.37)	41.85	50.36 (11.69)	26.98	48.42 (12.77)	22.88	50.12 (9.39)	31.35
42	40	51.88 (9.38)	33.11	56.58 (4.95)	46.69	36.92 (12.80)	11.31	51.58 (8.86)	33.86	51.00 (9.49)	32.03
48	40	50.50 (12.39)	25.71	57.25 (7.16)	42.94	29.62 (14.97)	--	40.38 (12.37)	15.63	47.75 (13.06)	21.64
54	40	53.90 (9.71)	34.47	58.29 (4.27)	49.75	32.18 (17.57)	--	29.27 (13.49)	2.29	48.88 (11.01)	26.86
60	40	48.50 (9.95)	28.60	59.25 (2.67)	53.92	32.63 (18.64)	--	31.13 (12.06)	7.00	55.00 (5.88)	43.73

**Table 3:** Mean scores: comparative results for five countries

TP	Sample	N	Communication		Gross motor		Fine motor		Problem-solving		Social-personal	
			Mean	*P*-Value	Mean	*P*-Value	Mean	*P*-Value	Mean	*P*-Value	Mean	*P*-Value
*4*	US	1380	51 (9.00)	0.00*	55 (7.00)	0.00*	49 (11.00)	0.03*	53 (9.00)	0.59	51 (9.00)	0.18
	Norway	176	50 (8.00)	0.00*	55 (7.00)	0.00*	50 (11.00)	0.14	55 (6.00)	0.12	50 (10.00)	0.07
	Korea	99	51.9 (8.40)	0.00*	50.9 (11.10)	0.00*	45.8 (13.97)	0.00*	52.9 (10.99)	0.68	48.6 (11.10)	0.02*
	Spain	NA	NA	NA	NA	NA	NA	NA	NA	NA	NA	NA
	S.A.	38	57.25 (5.66)		58.59 (4.13)		52.63 (9.47)		52.00 (11.37)		52.89 (8.43)	
*8*	US	1285	54 (9.00)	0.03*	50 (13.00)	0.00*	54 (9.00)	0.00*	52 (10.00)	1	51 (11.00)	0.14
	Norway	165	53 (7.00)	0.00*	47 (13.00)	0.00*	56 (7.00)	0.19	52 (8.00)	1	51 (8.00)	0.16
	Korea	82	43.66 (10.50)	0.00*	48.35 (12.65)	0.00*	46.95 (14.50)	0.00*	47.5 (10.50)	0.06	47.56 (11.15)	0.00*
	Spain	NA	NA	NA	NA	NA	NA	NA	NA	NA	NA	NA
	S.A.	40	56.13 (5.83)		55.00 (8.16)		57.50 (6.20)		52.00 (12.75)		53.25 (9.23)	
*12*	US	1091	42 (13.00)	0.00*	49 (15.00)	0.27	49 (10.00)	0.01*	49 (12.00)	0.79	45 (13.00)	0.64
	Norway	145	42 (14.00)	0.00*	46 (16.00)	0.03*	52 (9.00)	0.00*	51 (10.00)	0.46	44 (12.00)	0.96
	Korea	124	43.32 (13.68)	0.00*	51.64 (13.82)	0.92	48.79 (13.43)	0.05*	47.88 (14.19)	0.47	41.28 (13.75)	0.33
	Spain	34	39 (13.00)	0.00*	40 (20.00)	0.01*	46 (14.00)	0.64	49 (11.00)	0.85	38 (14.00)	0.08
	S.A.	40	55.50 (6.68)		51.38 (13.2)		44.63 (10.58)		49.50 (11.65)		43.88 (14.70)	
*16*	US	976	49 (12.00)	0.49	55 (12.00)	0.25	52 (11.00)	0.42	50 (11.00)	0.38	48 (11.00)	0.00*
	Norway	146	42 (13.00)	0.01*	57 (9.00)	0.03*	54 (8.00)	0.07	54 (9.00)	0.18	48 (10.00)	0.00*
	Korea	128	38.05 (15.22)	0.00*	54.66 (13.62)	0.42	45.73 (16.09)	0.04*	46.64 (15.42)	0.03*	44.14 (13.31)	0.00*
	Spain	34	38 (13.00)	0.00*	52 (10.00)	0.68	45 (8.00)	0.02*	46 (10.00)	0.02*	46 (13.00)	0.01*
	S.A.	40	47.63 (12.30)		53.00 (10.49)		50.50 (11.37)		51.50 (10.51)		52.75 (7.68)	
*20*	US	845	48 (11.00)	0.44	55 (10.00)	0.57	54 (7.00)	0.00*	49 (10.00)	0.00*	53 (9.00)	0.65
	Norway	138	47 (15.00)	0.74	57 (6.00)	0.07	52 (9.00)	0.00*	50 (9.00)	0.00*	51 (9.00)	0.11
	Korea	144	39.27 (16.38)	0.02*	55.76 (8.84)	0.33	45.35 (11.66)	0.58	45.38 (12.30)	0.02*	50.35 (10.12)	0.05*
	Spain	56	38 (16.00)	0.01*	52 (12.00)	0.32	50 (10.00)	0.01*	44 (11.00)	0.16	47 (12.00)	0.00*
	S.A.	41	46.10 (15.39)		54.15 (9.35)		44.15 (12.14)		40.73 (11.16)		53.66 (9.15)	
*24*	US	820	50 (11.00)	0.85	54 (9.00)	1	53 (8.00)	0.00*	51 (10.00)	0.02*	52 (8.00)	0.14
	Norway	128	53 (10.00)	0.23	56 (6.00)	0.3	53 (8.00)	0.00*	50 (9.00)	0.06	51 (8.00)	0.39
	Korea	144	48.92 (14.49)	0.53	55.28 (9.96)	0.53	48.33 (10.45)	0.08	48.75 (11.95)	0.2	48.54 (11.26)	0.68
	Spain	56	44 (17.00)	0.04*	50 (12.00)	0.1	53 (11.00)	0.00*	48 (NA)	NA	48 (10.00)	0.54
	S.A.	39	50.38 (12.42)		54.00 (11.39)		44.13 (13.58)		45.75 (13.04)		49.36 (10.83)	
*30*	US	562	56 (9.00)	0.00*	51 (10.00)	0.00*	50 (12.00)	0.06	51 (11.00)	0.00*	53 (8.00)	0.00*
	Norway	134	57 (7.00)	0.00*	56 (6.00)	0.93	50 (13.00)	0.08	52 (9.00)	0.00*	53 (7.00)	0.00*
	Korea	223	53.21 (12.92)	0.04*	53.68 (12.43)	0.2	49.53 (14.33)	0.1	51.05 (13.69)	0.00*	49.84 (12.25)	0.08
	Spain	86	54 (10.00)	0.02*	53 (8.00)	0.1	53 (11.00)	0.01*	49 (NA)	NA	51 (8.00)	0.02*
	S.A.	40	49.15 (11.17)		55.87 (9.40)		45.12 (15.59)		41.71 (13.26)		46.87 (9.32)	
*36*	US	512	54 (8.00)	0.38	55 (10.00)	0.2	52 (11.00)	0.39	55 (8.00)	0.00*	53 (7.00)	0.06
	Norway	126	54 (7.00)	0.41	56 (7.00)	0.65	52 (10.00)	0.42	54 (9.00)	0.01*	53 (8.00)	0.08
	Korea	226	54.82 (10.51)	0.83	55.2 (10.85)	0.31	53.25 (11.02)	0.15	53.24 (10.51)	0.03*	50.11 (10.68)	1
	Spain	70	54 (9.00)	0.49	52 (11.00)	0.01*	54 (9.00)	0.09	48 (NA)	NA	51 (9.00)	0.63
	S.A.	41	55.12 (7.70)		56.59 (7.37)		50.36 (11.69)		48.42 (12.77)		50.12 (9.39)	
*48*	US	336	56 (9.00)	0.01*	52 (10.00)	0.00*	44 (14.00)	0.00*	57 (8.00)	0.00*	49 (13.00)	0.57
	Norway	100	56 (6.00)	0.01*	54 (9.00)	0.03*	50 (13.00)	0.00*	54 (9.00)	0.00*	56 (7.00)	0.00*
	Korea	224	52.55 (9.70)	0.33	52.5 (8.25)	0.00*	51.09 (10.00)	0.00*	52.05 (8.67)	0.00*	53.86 (7.29)	0.01*
	Spain	NA	NA	NA	NA	NA	NA	NA	NA	NA	NA	NA
	S.A.	40	50.50 (12.39)		57.25 (7.16)		29.62 (14.97)		40.38 (12.37)		47.75 (13.06)	
*60*	US	125	50 (9.00)	0.4	52 (10.00)	0.00*	51 (10.00)	0.00*	51 (11.00)	0.00*	54 (7.00)	0.38
	Norway	82	55 (5.00)	0.00*	55 (6.00)	0.00*	51 (10.00)	0.00*	52 (9.00)	0.00*	56 (7.00)	0.41
	Korea	321	50.64 (10.10)	0.21	53.18 (9.63)	0.00*	52.65 (9.61)	0.00*	55.05 (9.19)	0.00*	54.06 (7.91)	0.36
	Spain	NA	NA	NA	NA	NA	NA	NA	NA	NA	NA	NA
	S.A.	40	48.50 (9.95)		59.25 (2.67)		32.63 (18.64)		31.13 (12.06)		55.00 (5.88)	

*p < 0.05

**Table 4:**
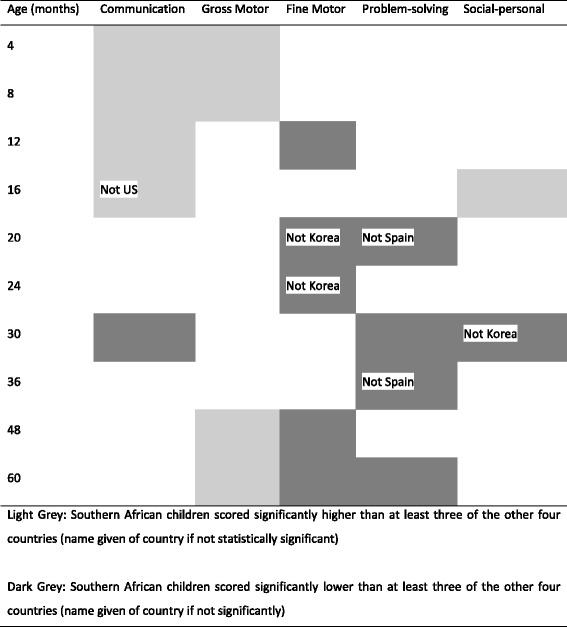
Summary of difference between SA and five comparison countries

Light Grey: Southern African children scored significantly higher than at least three of the other four countries (name given of country if not statistically significant)

Dark Grey: Southern African children scored significantly lower than at least three of the other four countries (name given of country if not significantly)

### Communication

Communication scores were found to be significantly higher among Southern African (SA) children than comparison countries from 4 to 16 months of age (*p* < 0.00 USA, *p* < 0.00 Norway, *p* < 0.00 Spain). These higher scores are replaced by non-significant differences between SA and other children at 20, 24, 36, 48 and 60 months of age. There is an outlier at 30 months during which time SA children score worse than their peers in all four of comparison countries.

### Gross motor

SA children were found to score significantly higher than their peers in this domain at both ends of the measured range. Significantly higher scores were seen for SA children at 4, 8, 48 and 60 months. At four months SA children had a mean score of 58.59 (sd 4.13) compared to a mean of 55.00 (7.00) for the US, 55.00 (7.00) Norway and 50.90 (11.10) for Korea. At 60 months, SA children had a mean score of 59.25 (s.d. 2.67) compared to a mean of 52.00 (10.00) for the US, 55.00 (6.00) Norway and 53.18 (9.63) for Korea.

### Fine motor

SA children performed significantly lower than children from other countries at many of the measured time points after 12 months. At 5 out of the 8 measured ages (12, 20, 24, 48 and 60), SA children scored significantly lower than children in at least three of the four comparison countries. SA children scored significantly lower on the fine motor sub-scale than all comparison countries at 12, 48 and 60 months. As found within the gross motor domain, SA children do, however, perform significantly better than the US and Korean children at both 4 and 8 months on the fine motor items.

### Problem solving

Problem solving items produced a number of significantly lower scores for older SA children as compared to the comparison countries. At months 20 (mean - 40.73, s.d. - 11.16), 30 (41.71, 13.26), 36 (48.42, 12.77) and 60 (31.13, 12.06), SA children had significantly lower scores. The higher scores observed in SA children on communication, gross motor and fine motor at months 4 and 8 were not found in these ages on this domain.

### Social-personal

On the whole SA children performed about as well as children from comparison countries across the entire age range. SA children did significantly better at 16 months and significantly worse at 30 months but the overall trend was similar.

## Discussion

The earliest written observations of African children’s development made by European explorers, which was coloured by their colonial mind set, tended to emphasise things that African children couldn’t or didn’t do when compared to European children, or things that African parents didn’t do in the same way that European parents did. As a consequence, many of the practices of African families that promote young children’s development were not recorded until the late 1950s. These practices were highlighted by psychologists and health practitioners working in Africa, who found that African children in their first year of life scored higher than American and European children when assessed on standardised tests of development [22, 23].

At first, this so called *African infant precocity* was ascribed to longer gestation, a more robust motor system, and perhaps an evolutionary adjustment to harsher conditions and more physical exercise. But later, as a result of detailed studies, it was suggested that many of the day-to-day practices of African mothers promote and support the development of young infants, and that these practices may account for their children’s faster development in the first year of life [24, 25]. Some such common practises include carrying the baby on the mother’s body, feeding on demand, immediately pacifying babies when they cry, mother and baby sleeping together, and the exposure of babies to a wide social network of kin and family [26–29].

These data paint a similar picture with around half of all age/domain comparisons showing Southern African children to be scoring similarly to at least two of the four comparison countries. Southern African children score particularly high on the communication and gross motor domains with better scores overall in the first 12 to 16 months of life. Where differences did occur they were primarily in the older age ranges or in the domains of fine motor and problem-solving.

These mean differences may result from a number of factors. First, each domain may be most susceptible to interpretation based on differences in cultural understanding and expectations. For example, although parents from many different cultures play some variant of the “peek-a-boo” game, it may not be called this in all contexts. Without adequate cultural adaptation it may appear that caregivers and children are unable to complete this item [30]. Second, as indicated above, common cultural practices may enhance or hinder children’s ability to complete some of the items in the ASQ. For example, and as mentioned previously, all over Africa, women carry babies in blankets, towels or cloth slings on their back, at their side or on their front. They carry their babies while they walk, talk and do their work in the fields and around the house. This constant close proximity helps babies feel secure and the rhythm of the mothers walking and movements are calming; at the same time, the upright position helps the baby to be alert to the many sights and sounds to which the baby is exposed as a result of the mother’s activities. The mother is very sensitive to the baby’s state because the baby’s body is against hers. She can feel when her baby is fretting, or wants to urinate, and she can respond immediately to her baby. This practise and its consequences may be partially responsible for some of the early advantage seen in the domains of communication and gross motor development [22–24, 31].

Similarly, cultural practices and social context may partly explain some of the lower scores achieved by children in this sample. For example, many large southern African families living with resource constraints require older children to be responsible for the care of younger children. This may mean that a primary school aged girl takes on this responsibility. Such a young child may be unable to stimulate a toddler or provide opportunities for learning at the level of an adult caregiver [32]. Finally, test performance in the older age ranges can be influenced by the availability and quality of learning materials at home and in preschools. As presented by Hsiao et al.[18] (submitted), as many as half of the items in the problem solving domain have a poor pass rate, covering concepts taught in preschool programmes such as colour identification, counting, number recognition, recognizing the letters in one’s own name, and so on.

These findings, supplemented by the companion work of Hsiao et al. [18] (submitted), suggest that despite the differences found, the ASQ-3 is a developmental screening tool that stands up fairly well to the cultural and contextual differences of southern African children, particularly in the first 2 years of life. Hsiao et al. [18] provide specific recommendations regarding the use of the ASQ in the light of our findings. Firstly, that the ASQ provides a good overall assessment of children’s developmental status up until the end of the fourth year of life. From 48 months onwards, southern African children are prejudiced by their lack of exposure to concepts commonly taught in preschools. However, items at the older ages can be used as a criterion-referenced tool to ascertain what preschool concepts children have learned.

Despite the support these data provide for the routine use of the ASQ as a developmental screening instrument in Southern Africa, it is not recommended for clinical or individual assessment of children. Children who screen positive for developmental delay must be referred for a more thorough assessment by an early child development specialist to validate the screen and establish what intervention is the best course of action. Early identification and accurate assessment is an important starting point to better understand and anticipate the needs of children and their link to services. Without a strong commitment to these goals, millions of children in low- and middle-income countries will continue to lag behind their peers in high income countries as they fail to reach their social, cognitive and physical developmental potential [3].

Given the scarcity of trained professionals to screen young children for developmental delays in low- and middle-income countries [33], this body of work in adapting and evaluating the ASQ, has demonstrated the feasibility of using the instrument in developing country settings and at the same time, actively involving caregivers as key partners in assessing and planning interventions for their children.

### Strengths and limitations

The current study collected unique data, being the first to compare performance on the ASQ-3 by children in Southern Africa to children from other countries around the world. A second strength is that the study looks at children’s performance across all 21 ages of the ASQ-3. However, a limitation of the study is that the sample size is smaller than in the comparison studies, with only 40 children in each of the age categories. Another limitation of the design is that it only sampled children with a low risk of developmental delay, although all children lived in contexts of high poverty and social deprivation.

## Conclusion

This study provides further support for the feasibility of using the ASQ, culturally adapted and translated, in low- and middle-income countries, with some caveats regarding items assessing specific school-readiness concepts. Our results also confirm the early developmental advantage of young children in southern Africa across a number of developmental domains. However, as has been found in other studies, this early advantage is eroded after the first year and their development trajectories are significantly worse than their peers in other parts of the world by the age of 5 years. The decline is frequently attributed to increased exposure to infections when children become mobile in makeshift settlements, poorer nutrition as children are expected to be more independent with respect to self-feeding, lack of language and learning stimulation in deprived households when adults labour for long hours in workplaces far from home, and lack of quality childcare and preschool experience. With early and appropriate intervention, this head start could potentially be protected and maintained to ensure improved early learning and school readiness among children living in southern Africa.

## Abbreviations

ASQ-3: Ages and Stages Questionnaire version 3
